# Clinical evidence of acupuncture and related therapy in patients with cancer-pain: A protocol for systematic review and meta-analysis

**DOI:** 10.1097/MD.0000000000023119

**Published:** 2020-11-06

**Authors:** Jingchun Zeng, Runjin Zhou, Zhenke Luo, Na Zhang, Zijun Liu, Guohua Lin

**Affiliations:** a Department of Acupuncture, The First Affiliated Hospital, Guangzhou University of Chinese Medicine; b Guangzhou University of Chinese Medicine, Guangzhou, People's Republic of China.

**Keywords:** acupuncture and related therapy, cancer pain, high quality RCTs, protocol, protocol, systematic review

## Abstract

**Background:**

Cancer pain (CP) is one of the common complications of cancer. During the treatment, oral medication, radiotherapy and chemotherapy bring certain adverse reactions to patients with CP; a safe way to treat this disease is necessary. Acupuncture and related therapies for CP with few side effects have been gradually accepted. But at present the evidence is insufficient, the related research is not thorough enough. The purpose of this study was to investigate the efficacy and safety of acupuncture and related therapies for CP.

**Methods:**

The Preferred Reporting Items for Systematic Review and Meta-Analysis Protocols (PRISMA-P) guidelines were used to design this protocol. The final study will also be conducted under the PRISMA guidelines for systematic reviews and meta-analysis. An electronic search will be conducted in Medline, Embase, and the Cochrane Central Register of Controlled Trials databases through January 2020. The search will be conducted in English. Risk of bias will be assessed by the Cochrane Collaboration tool and the collected evidence will be nar-ratively synthesized. We will also perform a meta-analysis to pool estimates from studies considered to be homogenous. Subgroup analyses will be based on intervention or overall bias. The strength of evidence will be evaluated by the Grading of Recommendations, Assessment, Development and Evaluation scale.

**Results:**

This systematic review will summarize high quality clinical evidence to assess and appraise the effectiveness and safety of acupuncture and related therapies for CP patients.

**Conclusion:**

The meta-analysis will assess evidence from randomized controlled trials of acupuncture and related therapies and CP types.

**INPLASY registration number:**

INPLASY202040129.

## 1 Introduction

There are 7 million new cases of cancer every year worldwide. Most cancer patients suffer from moderate to severe pain.^[1]^ Nearly 70% of patients with CP receive no effective analgesic treatment.^[2,3]^ In China, about 1 million people suffer from CP each year, and 60% to 90% of patients go to the hospital for it.^[4]^ These patients with CP also present insomnia, anxiety, depression, and other psychiatric symptoms.^[5,6]^ CP not only diminishes patients quality of life and physical function,^[7]^ but also incurs economic costs to both their families and to society.^[8]^

In addition to the invasion, compression, and metastasis of the cancer itself,^[9]^ the occurrence of CP is also related to examinations, radiation therapy, and chemotherapy.^[10,11]^ The three-step pain relief for CP proposed by the WHO has been widely used in clinical practice,^[12]^ but addiction to and tolerance of opioid analgesics such as morphine and oxycodone remain formidable problems.^[13]^ Acupuncture has a significant effect on acute and chronic pain.^[14]^ As an alternative therapy for CP, acupuncture and related therapies are widely used in China and even internationally.^[15,16]^ The National Adult Cancer Pain Comprehensive Cancer Network Guide states that acupuncture and related therapies may be important complements for CP analgesia.^[17]^ The proportion of CP among all forms of pain has also increased due to the increased incidence of cancer. Clinical trials have found that acupuncture and related therapies have several pain relieving effects, such as improving joint pain symptoms caused by aromatase inhibitors in patients with early breast cancer.^[15,16,18]^ Acupuncture combined therapy could reduce the intensity of CP, lighten the dose of oral opioids, and mitigate nausea and discomfort after radiotherapy.^[19–21]^ In addition to acupuncture, related therapies such as tui-na could also improve anxiety and mood disorders due to CP.^[22,23]^ Additionally, transcutaneous electrical acupoint stimulation (TEAS) can mitigate the nausea and vomiting caused by oral chemotherapy drugs.^[24]^ These treatments are more acceptable to patients as non-invasive interventions. However, research has been hindered by problems such as insufficient participants and inconsistent power indicators.^[25]^

This protocol outlines a systematic review and meta-analysis of the benefits of acupuncture and related therapies for patients with CP. The potential control groups are sham needle, analgesics, placebo, and a blank control group. The primary outcomes are the degree of pain relief, grade and dose changes in analgesics, and adverse events. This study will provide objective clinical evidence for the safety and effectiveness of acupuncture and related therapies (such as moxibustion, massage, and tui-na) for patients with CP.

## 2 Objectives

This protocol proposes a methodology for conducting a systematic review and meta-analysis to provide clinical evidence of the benefits of acupuncture and related therapy in patients with CP. This may provide the evidence and form a treatment recommendation for clinicians and researchers.

## 3 Methods and analysis

We will conduct our meta-analysis in line with the Cochrane Handbook for Systematic Reviews of Interventions^[26]^ and report this meta-analysis based on the Preferred Reporting Items for Systematic Reviews and Meta-analyses (PRISMA) guidelines.^[27]^ The protocol has been registered on the International Platform of Registered Systematic Review and Meta-Analysis Protocols (INPLASY), registration number is INPLASY 202090073.

### 3.1 Inclusion and exclusion criteria

#### 3.1.1 Type of studies

There is no limit to the treatment cycle and the type of CP, the systematic review will include high-quality RCTs in English that assess its effectiveness and safety in improving CP and reducing related symptoms. Exclusion criteria included non-RCTs, retrospective studies, review studies, case reports, animal experiments.

#### 3.1.2 Type of participants

Adult cancer patients (age> =18 years) of either sex: pain caused by the cancer itself (such as cancer progression), or cancer treatments (such as tests, chemotherapy, or, surgery). Unlimited participant ethnicity.

#### 3.1.3 Type of interventions

##### 3.1.3.1 Experimental interventions

Acupuncture and related therapies are defined as acupuncture techniques and meridian acupoint stimulation methods, including acupuncture, electroacupuncture, ear acupuncture, 5 element needle, wrist-ankle acupuncture, fire needle, moxibustion, acupoint acupressure, transcutaneous electrical acupoint stimulation (TEAS), tui-na, massage and combined interventions.

##### 3.1.3.2 Comparison interventions

Blank control, placebo control, sham acupuncture control, and analgesics control are the comparator interventions. The placebo will be a needle attached to the surface of the skin that does not penetrate the skin.^[28]^ Sham acupuncture is defined as placing the needle in an area near, but not at, the acupuncture point, or the electrical stimulation of the skin above local acupoints by electrodes attached to the skin.^[28]^ We will investigate the following comparisons:

1. The experimental group and the control group each had routine nursing, and the effect was evaluated either with or without acupuncture intervention.
2. Acupuncture and related therapies are only compared to placebo or sham treatment.
3. Acupuncture and related therapies plus active treatment or drug treatment, compared with active treatment or drug treatment.
4. Acupuncture and related therapies plus active treatment methods or drugs, compared with placebo or sham treatment plus active treatment methods or drugs.

#### 3.1.4 Type of outcome measures

Pain relief as a curative effect, defined by original studies. The primary outcome is the change in grade and dosage of analgesics for CP. Secondary outcomes are quality of life and anxiety score.

### 3.2 Search strategy

#### 3.2.1 Electronic searches

Published RCTs will be retrieved by searching the Central Registers of Medline, Embase, and Cochrane Controlled Trials. We will use the following search terms: “randomized controlled trial” AND “cancer pain” AND “acupuncture” OR “electroacupuncture” OR “ear acupuncture” OR “5 element needle” OR “wrist-ankle acupuncture” OR “fire needle” OR “moxibustion” OR “acupoint acupressure” OR “transcutaneous electrical acupoint stimulation” OR “TEAS” OR “tui-na” OR “massage”. Languages will be restricted to English. See Table 1 for the search strategy.

**Table 1:** Search strategy used in the PubMed database.

MEDLIN, EMBASE, Cochrane Search strategy
1. Acupuncture
2. Acupuncture Therapy
3. meridian point^∗^ or meridian^∗^ or (ching adj2 lo) or (jing adj2 luo) or jingluo or acu point^∗^ or acu-point^∗^ or acupoint^∗^
4. acupuncture or electroacupuncture or electro-acupuncture or electro acupuncture or Zhenjiu or Zhenci or Cizhen or Dianzhen
5. acupressure^∗^ or acup^∗^ point^∗^ or mox^∗^ or needle^∗^ or auriculo-acup^∗^ or cup^∗^ or bloodlet^∗^
6. hot point^∗^
7. fire needle
8. Moxibustion
9. moxibustion$ or moxabustion$ or moxa$ or artemisia$ or mugwor$
10. transcutaneous electrical acupoint stimulation
11. TEAS
12. reflexology
13. massage
14. shiatsu or tui na in clinical trials
15. five element needle
16. wrist-ankle acupuncture
17. 1 or 2-16
18. CANCER$
19. NEOPLASMS$
20. TUMO^∗^R
21. ONCOLO$
22. CARCINOMA$
23. MALIGNAN$
24. 18 or 19-23
25. PAIN^∗^
26. 17 and 24 and 25
27. randomi?ed
28. randomized controlled trial
29. controlled clinical trial
30. placebo
31. clinical trials
32. randomly
33. trial
34. 27 or 28-33
35. 26 and 34
36. limit 35 to human [Limit not valid in CCTR; records were retained]

#### 3.2.2 Searching other sources

Similar meta-analyses have been published, with references to neuropathic pain textbooks, alternative and complementary medicine textbooks, and clinical guidelines for related trials set by the WHO's International Clinical Trials Registry Platform (ICTRP).

### 3.3 Data collection and analysis

#### 3.3.1 Selection of studies

**Figure 1:**
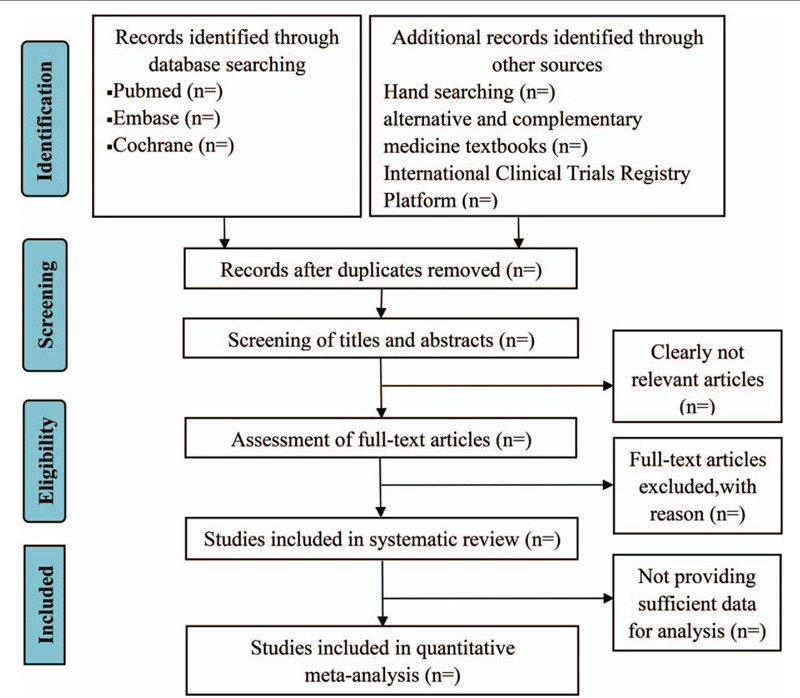
PRISMA flow chart of study process.

Studies will be screened by 2 independent researchers (RJZ and NZ) who are familiar with the literature management tools. The 2 authors measurement consistency in making simple inclusion/exclusion decisions will be calculated with Kappa Statistics. Kappa values between 0.40 to 0.59 are considered consistent, between 0.60 to 0.74 are fairly good, and 0.75 and above are very good.^[29]^ The 2 authors will review and screen the titles and abstracts to identify eligible trials. According to the inclusion criteria, duplicates will be removed with EndNote (v.x9.0). If necessary, the full text will be read, the exclusion studies will be listed in a table, and the reasons for the exclusion, and any differences, will be discussed with a methodology expert. See Figure 1 for the study flow diagram.

#### 3.3.2 Data extraction and management

Both authors (RJZ and ZKL) will use the data extraction form to extract the participants, and the following information will be extracted according to the CONSORT statement format: general information (year of publication, author, title, abstract, registration number, funding), research methods (research purpose, trial design, subjects, interventions, outcomes, randomization, blinding), results (number of randomized cases, subjects, baseline data, number of included cases, outcomes, and adverse reactions). We will enter the data into Review Manager (RevMan V.5.3). If necessary, we will contact the authors of the included studies for missing information or for clarification.

#### 3.3.3 Assessment of risk of bias in included studies

The risk of bias in 6 areas (sequence generation, allocation hiding, blindness, incomplete data evaluation, selective results reporting, and other sources of bias) will be assessed with the Cochrane Deviation Risk Collaborative Tool^[26]^ by 2 reviewers (RJZ and NZ). This will provide reasons to judge potential risk. Any disagreement will be resolved by discussion with a methodology expert (JCZ).

#### 3.3.4 Measures of treatment effect

A risk ratio (RR) based on a 95% CI will represent the evaluation of the effect of the dichotomous results. For continuous results, we will represent the estimated effect as the average difference within a 95% CI. The weighted mean difference will be used for data measured at the same scale and using the same units; otherwise, a standardized mean difference will be used.

#### 3.3.5 Unit of analysis issue

Based on the results, the change in grade and dosage of analgesics for CP will be aggregated and secondary results will be analyzed separately, including quality of life and anxiety score.

#### 3.3.6 Dealing with missing data

In cases of insufficient data from an experiment, we will try to contact the first author or the corresponding author of the included study. We will also use library resources to manually retrieve missing or insufficient data. If possible, an intent-to-treat analysis should be conducted including data for all participants in the group to which they were initially randomly assigned. Additionally, a sensitivity analysis should be conducted to determine whether the results are consistent.

#### 3.3.7 Assessment of heterogeneity

We will use *I*^2^ statistics to quantify the inconsistencies between the included studies. When an *I*^2^ value is less than 50%, the study will not be considered heterogeneous; an *I*^2^ value exceeding 50% indicates significant statistical difference between the trials. Qualitative, subgroup analysis will be performed to explore possible causes.

#### 3.3.8 Assessment of reporting biases

Bias and small-scale studies will be detected with funnel plots. If the meta-analysis includes more than 10 studies, the asymmetry will be explained with the Egger method.^[30]^

#### 3.3.9 Data synthesis

If a meta-analysis is possible, the results of the binary data will be expressed as RR using RevMan V.5.3, and the results will be expressed as standardized mean difference (SMD) for continuous data. If *I*^2^ test results are less than 50%, the data will be synthesized using a fixed effects model. If they are between 50% and 75%, the data will be synthesized using a random effects model. If they are over 75%, we will investigate possible causes from a clinical and methodological perspective and perform a subgroup analysis.

#### 3.3.10 Subgroup analysis

Subgroup analysis will be based on intervention (acupuncture, electroacupuncture, ear acupuncture, 5 element needle, wrist-ankle acupuncture, fire needle, moxibustion, acupoint acupressure, transcutaneous electrical acupoint stimulation (TEAS), tui-na, massage) or overall bias. The severity of the CP will also be considered.

#### 3.3.11 Sensitivity analysis

We will conduct a sensitivity analysis to verify the robustness of the research conclusions, assess the methodological quality, the study design, the effect of sample size and missing data, and the effect of the analysis method on the results of this review. The meta-analysis will be repeated, and lower quality studies will be excluded. These results will then be compared and discussed.

#### 3.3.12 Ethics and dissemination

This study does not require formal ethics approval, as the data to be used is not personal data and does not involve privacy. This article will evaluate the effects of acupuncture and related therapies on CP. Results will be disseminated through peer-reviewed publications or at relevant conferences.

## 4 Discussion

A meta-analysis has shown that acupuncture can relieve cancer-related pain, especially pain caused by malignant tumors and surgery.^[31]^ However, a Cochrane systematic review concluded that there is insufficient evidence that acupuncture relieves CP in adults.^[25]^ Existing meta-analyses have focused on acupunctures effect on CP.^[32,33]^ However, acupuncture-related therapies have yet to be summarized. In view of the current situation, a comprehensive meta-analysis is needed to evaluate the benefits of acupuncture and related therapies for CP. This study covers all RCTs of CP treated by acupuncture, and RCTs of most acupuncture-related therapies, so as to provide evidence for acupuncture and related therapies. This study has 2 primary limitations: It has a large sample size and diverse treatment methods, and only includes English literature. Additionally, the diverse evaluation criteria from various acupuncture and related therapies may have produced a high degree of heterogeneity.

## Author contributions

Guohua Lin is the guarantor of this article and will act as an arbitrator in the event of a dispute. RJZ and ZKL have established search strategies. RJZ, NZ and ZKL will independently complete the research selection, data extraction and assessment of the risk of bias. RJZ, NZ and ZJL will perform data synthesis. Subsequent and final versions of the plan have been rigorously reviewed, modified, and authorized by all authors.

**Conceptualization**: Guohua Lin, Jingchun Zeng.

**Data curation**: Runjin Zhou, Na Zhang, Zhenke Luo.

**Formal analysi**s: Runjin Zhou, Na Zhang, Zijun Liu.

**Funding acquisitio**n: Guohua Lin, Jingchun Zeng.

**Investigation**: Runjin Zhou, Zhenke Luo, Na Zhang.

**Methodology**: Jingchun Zeng, Zijun Liu.

**Project administration**: Guohua Lin, Jingchun Zeng.

**Resources**: Jingchun Zeng.

**Software**: Runjin Zhou, Na Zhang.

**Supervision**: Guohua Lin, Jingchun Zeng.

**Validation**: Zijun Liu, Jingchun Zeng.

**Visualization**: Runjin Zhou, Na Zhang, Zijun Liu, Jingchun Zeng.

**Writing – original draf**t: Runjin Zhou, Zhenke Luo.

**Writing – review & editing**: Guohua Lin, Jingchun Zeng.
